# The Paraventricular Nucleus of the Thalamus Is an Important Node in the Emotional Processing Network

**DOI:** 10.3389/fnbeh.2020.598469

**Published:** 2020-10-29

**Authors:** Jessica R. Barson, Nancy R. Mack, Wen-Jun Gao

**Affiliations:** Department of Neurobiology and Anatomy, Drexel University College of Medicine, Philadelphia, PA, United States

**Keywords:** anterior, anxiety, arousal, depression, fear, posterior, reward, stress

## Abstract

The paraventricular nucleus of the thalamus (PVT) has for decades been acknowledged to be an important node in the limbic system, but studies of emotional processing generally fail to incorporate it into their investigational framework. Here, we propose that the PVT should be considered as an integral part of the emotional processing network. Through its distinct subregions, cell populations, and connections with other limbic nuclei, the PVT participates in both major features of emotion: arousal and valence. The PVT, particularly the anterior PVT, can through its neuronal activity promote arousal, both as part of the sleep-wake cycle and in response to novel stimuli. It is also involved in reward, being both responsive to rewarding stimuli and itself affecting behavior reflecting reward, likely *via* specific populations of cells distributed throughout its subregions. Similarly, neuronal activity in the PVT contributes to depression-like behavior, through yet undefined subregions. The posterior PVT in particular demonstrates a role in anxiety-like behavior, generally promoting but also inhibiting this behavior. This subregion is also especially responsive to stressors, and it functions to suppress the stress response following chronic stress exposure. In addition to participating in unconditioned or primary emotional responses, the PVT also makes major contributions to conditioned emotional behavior. Neuronal activity in response to a reward-predictive cue can be detected throughout the PVT, and endogenous activity in the posterior PVT strongly predicts approach or seeking behavior. Similarly, neuronal activity during conditioned fear retrieval is detected in the posterior PVT and its activation facilitates the expression of conditioned fear. Much of this involvement of the PVT in arousal and valence has been shown to occur through the same general afferents and efferents, including connections with the hypothalamus, prelimbic and infralimbic cortices, nucleus accumbens, and amygdala, although a detailed functional map of the PVT circuits that control emotional responses remains to be delineated. Thus, while caveats exist and more work is required, the PVT, through its extensive connections with other prominent nuclei in the limbic system, appears to be an integral part of the emotional processing network.

## Introduction

While the paraventricular nucleus of the thalamus (PVT) has for decades been acknowledged to be an important node in the limbic system (see, for example, Jayaraman, 1985; Su and Bentivoglio, 1990; Hsu et al., 2014; Colavito et al., 2015; Kirouac, 2015), studies of emotional processing, defined here as the process by which emotions are generated in response to specific stimuli, generally fail to incorporate it into their investigational framework. Here, we propose that the PVT should be considered as an integral part of the emotional processing network. According to the Two-Dimensional Theory of Emotion (Lang, 1995), affective responses can be qualified according to their placement along two axes: (1) arousal, reflecting the intensity of the stimulus; and (2) valence, reflecting the hedonic value of the stimulus. Under this framework, the PVT can be considered to participate in both major features of emotion, arousal, and valence. Thus, it is both responsive to and also influences not just arousal but also reward, motivation, depression, anxiety, stress, and fear (see below for details), generating emotional states and translating them into behavioral responses. It is involved in both conditioned responses, which require learning, and also unconditioned or primary emotional responses. Just as arousal and valence reflect two distinct dimensions of affect, however, the participation of the PVT in these dimensions may originate from different subdivisions of the PVT. Thus, the main purpose of this review is to illustrate the multiple ways in which the PVT participates in emotional processing, and also to address, where known, which specific subregions and cell populations of the PVT contribute to each facet of this phenomenon.

We note here that while emotionally laden stimuli can generate motivated behavior that is directed toward or away from those stimuli and, as such, motivated behavior can be difficult to disentangle from affective behavior, our specific focus here is on emotional processing. A growing body of literature, however, has demonstrated that the PVT also plays an integral role in motivated behavior, particularly motivated behavior that is linked to drugs of abuse. For more information on the involvement of the PVT in motivated behavior, the reader is directed to several excellent reviews (Kirouac, 2015; Millan et al., 2017; Matzeu and Martin-Fardon, 2018; Zhou and Zhu, 2019).

## Anatomical Characteristics of the PVT

In the rodent, the PVT, a prominent nucleus in the dorsal midline thalamus that is positioned just ventral to the dorsal third ventricle, extends through a relatively long rostrocaudal axis (more than 3.2 mm in the adult rat and 2.1 mm in the adult mouse; Paxinos and Franklin, 2004; Paxinos and Watson, 2005). It is composed of at least two discrete clusters of cells, which were first distinguished by Gurdjian in 1927 as the *nucleus paraventricularis anterior* and *nucleus paraventricularis posterior* (Gurdjian, 1927). While many laboratories continue to separate the PVT into rostral and caudal halves, atlases of the rodent brain often differentiate between anterior PVT, PVT (or middle PVT), and posterior PVT (e.g., Paxinos and Franklin, 2004; Paxinos and Watson, 2005). Thus, careful attention should be paid when generating conclusions from the literature on this brain region. In this review, we distinguish between the three subregions (anterior, middle, and posterior PVT) whenever publications have explicitly made this distinction or have provided anatomical coordinates in such a way that the subregion can be determined.

As a whole, the PVT has extensive connections with the rest of the limbic system. It receives afferent inputs from multiple brain regions that process a variety of information, including defensive, visceral, nociceptive, gustatory, circadian, and executive function (Kirouac, 2015). For example, it receives serotonin from the dorsal and median raphe nuclei (Otake et al., 1995); norepinephrine from the locus coeruleus, reticular formation, and nucleus of the solitary tract (Phillipson and Bohn, 1994; Otake et al., 1995); dopamine from the hypothalamus and periaqueductal gray (Li S. et al., 2014); corticotropin-releasing factor from the amygdala and bed nucleus of the stria terminalis (BNST; Otake and Nakamura, 1995); and orexin/hypocretin from the hypothalamus (Peyron et al., 1998). In turn, it sends glutamatergic and peptidergic efferent projections to various limbic regions (Arluison et al., 1994; Csáki et al., 2000), most densely to the nucleus accumbens (Parsons et al., 2007; Dong et al., 2017), but also the BNST (Dong et al., 2017), central nucleus of the amygdala (Li and Kirouac, 2008; Dong et al., 2017), prefrontal cortex (Huang et al., 2006), and hypothalamus (Csáki et al., 2000). Of note, a specific investigation of projections from the PVT to the nucleus accumbens, BNST, and central nucleus of the amygdala has found a moderate-to-high level of collateralization (Dong et al., 2017), suggesting potential coordination in its efferent output.

**Figure 1:**
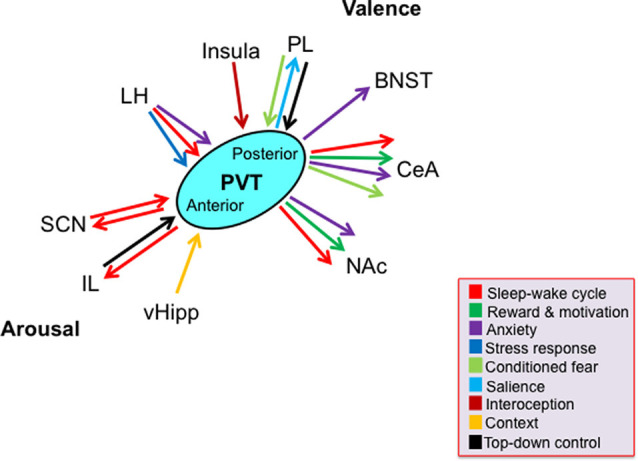
Schematic representing key limbic brain regions and associated functions by which the paraventricular nucleus of the thalamus, across its antero-posterior axis, contributes to the two dimensions of emotion: arousal and valence. Arrows denote function(s) associated with the projections according to their color. Most, but not all, depicted functions have been directly tested, as discussed in the text. Abbreviations: BNST, bed nucleus of the stria terminalis; CeA, central nucleus of the amygdala; IL, infralimbic cortex; LH, lateral hypothalamus; NAc, nucleus accumbens; PL, prelimbic cortex; PVT, paraventricular nucleus of the thalamus; SCN, suprachiasmatic nucleus; vHipp, ventral hippocampus.

While the rodent PVT subregions share many of the same afferents and efferents with each other, there are significant and notable differences in the density of these projections (Figure 1). For example, compared to inputs from other cortical regions, the anterior half of the PVT receives greater inputs from the ventral hippocampal subiculum and infralimbic cortex that convey information about motivational state and arousal, respectively. In contrast, compared to inputs to the anterior PVT, the posterior half of the PVT receives greater inputs from the prelimbic, infralimbic, and anterior insular cortices, that provide information about executive function, taste, and visceral sensation (Li and Kirouac, 2012; Kirouac, 2015). Moreover, while the entire PVT receives dense projections of orexin from the hypothalamus, which coveys information about arousal and stress, the posterior PVT receives heavier orexin innervation than the anterior PVT (Kirouac et al., 2005). Conversely, the anterior PVT projects widely to limbic areas, with denser projections to the suprachiasmatic nucleus (SCN), which is associated with circadian rhythm, while the more restricted projections of the posterior PVT are heavier to areas of the extended amygdala, including the BNST and central nucleus of the amygdala, which are involved in anxiety and fear (Moga and Moore, 1997; Li and Kirouac, 2008; Vertes and Hoover, 2008; Dong et al., 2017). While most neurons across the PVT project to the nucleus accumbens and a proportion of these provide collateral innervation of the BNST and central nucleus of the amygdala, the anterior PVT sends more projections to the dorsomedial accumbens shell, associated with appetitive behaviors, while the posterior PVT sends more projections to the ventromedial accumbens shell, associated with aversive behaviors (Dong et al., 2017). Moreover, efferent fibers have been found to travel from the anterior PVT to the posterior PVT but have not been identified in reverse, indicating that information flow may be unidirectional within the PVT (Vertes and Hoover, 2008). Together, these anatomical connections position the PVT to influence and coordinate affective behavioral responses, and they suggest that the anterior half of the PVT may have a somewhat more prominent role in arousal, while the posterior half of the PVT is more involved in valence.

Recent research has suggested that the divisions of the PVT may be more complex than previously described. Specifically, two well-defined neuronal subtypes, while largely restricted to the anterior and posterior halves of the PVT, respectively, demonstrate an antero-posterior gradient (Gao et al., 2020). Thus, while each subtype is most abundant in one half of the PVT, it is nonetheless also present in the other half, with the middle PVT subregion serving as a transition zone between the two neuronal subtypes (Gao et al., 2020). Similarly, of the PVT projections to the nucleus accumbens shell, the dorsomedial-projecting neurons show a progressive decrease from the anterior to the posterior PVT while the ventromedial-projecting neurons show the opposite gradient, decreasing from the posterior to the anterior PVT (Dong et al., 2017). These distribution gradients may explain historically conflicting findings regarding the responses and functional effects of cells throughout the PVT.

## Unconditioned Affective Behavior

In tests of unconditioned affective behavior, the PVT is involved in both arousal and valence, with changes in its neuronal activity responding to and influencing indicators of arousal, reward, depression, anxiety, and stress (see below for details). While the anterior and posterior halves of the PVT both appear to be involved in these behaviors, their relative contributions to each behavior demonstrate significant variation, suggesting that there may be a gradient of cells across the antero-posterior PVT axis that participate in them.

### Arousal—the PVT Regulates Arousal in a Subregion- and Cell Subpopulation-Specific Manner

The PVT shows a clear role in arousal, with slightly more evidence being generated from research investigating the anterior PVT than the posterior PVT. In work on circadian arousal, early research in rats demonstrated that levels of c-Fos (used as a marker for neuronal activity) are increased in the PVT in the dark (active) phase relative to the light phase (Peng et al., 1995), with levels peaking 4–6 h after lights-off (Mendoza et al., 2005; Ren et al., 2018). With a combination of c-Fos immunohistochemistry with retrograde tract-tracing, it has been shown that this increase occurs in both anterior PVT neurons projecting to the amygdala and posterior PVT neurons projecting to the nucleus accumbens (Peng et al., 1995). Moreover, with the preparation of slices during the dark cycle and examination using patch-clamp techniques, the anterior PVT is more likely to demonstrate spontaneous activity and increased depolarization compared to when it is prepared during the light (Kolaj et al., 2012), confirming that the anterior PVT is more active during the dark cycle. Recordings in mice have further clarified that population calcium activity in the PVT is greater during wakefulness than during sleep and that neuronal firing rate, as measured with electrophysiology, is also higher during wakefulness (Ren et al., 2018). Of note, during the transition from NREM sleep to wakefulness, terminals in the infralimbic cortex from galanin-containing PVT neurons, which in the mouse are denser in the anterior than the posterior PVT, demonstrate a decrease rather than an increase in activity, as measured by calcium transients (Gao et al., 2020). These findings suggest that cells within each PVT subregion may not uniformly demonstrate changes in activity in relation to the sleep-wake cycle; rather, specific populations of cells in the PVT are more active during behavioral arousal.

Beyond changing their activity during wakefulness compared to sleep, neurons in the PVT may themselves drive wakefulness. Investigation of population calcium activity in the mouse PVT shows that neuronal activity begins to increase before the onset of behavioral arousal (Ren et al., 2018). Further, optical stimulation of glutamatergic neurons in the PVT during NREM sleep promotes wakefulness, and this occurs *via* projections to the nucleus accumbens but not the prefrontal cortex and can be driven by orexin afferents from the lateral hypothalamus (LH; Ren et al., 2018). Conversely, chemogenetic inhibition, or lesioning of the mouse PVT decreases wakefulness during the dark phase of the light-dark cycle, when mice are typically more active (Ren et al., 2018). On the other hand, chemogenetic activation prior to dark onset of galanin-containing PVT neurons, which in the mouse are denser in the anterior PVT, decreases wakefulness and increases NREM sleep (Gao et al., 2020). Of note, in addition to its sizeable hypothalamic orexin input (Peyron et al., 1998), which can drive behavioral arousal (Ren et al., 2018), the PVT also receives extensive afferent input from the SCN (Novak et al., 2000; Peng and Bentivoglio, 2004), which regulates the circadian clock (Luppi and Fort, 2019). Thus, neuronal activity in the PVT can promote wakefulness and the PVT itself is positioned as a major recipient of circadian and arousal signaling.

The PVT is also involved in arousal in cases that are not dependent on the light-dark cycle. Population calcium signals in the mouse middle PVT are increased by a variety of novel or unfamiliar stimuli across a range of modalities, including olfactory, visual, and auditory (Zhu et al., 2018), indicating that the PVT responds to behaviorally relevant events. In rats maintained in constant darkness, levels of c-Fos in the PVT are increased at the time of scheduled access to a palatable meal (Mendoza et al., 2005), and in neonatal rabbits, levels of c-Fos in the PVT are increased during anticipatory arousal before scheduled maternal nursing (Allingham et al., 1998). This indicates that the PVT signals not just the response to a behaviorally relevant event but also the anticipation of one. While these studies do not differentiate between anterior and posterior subregions, they indicate that the PVT overall participates not just in arousal related to the sleep-wake cycle but also in arousal related to salient stimuli and events.

### Reward—the PVT Is Responsive to Reward Stimuli and Affects Reward-Related Behaviors

Research on reward suggests that the anterior portion of the PVT may be more involved in affecting reward-related behavior while the posterior PVT is more responsive to reward stimuli. An early indication for a role of the PVT in reward came in 1982, when Clavier and Gerfen demonstrated in male rats that intracranial self-stimulation could be supported by electrode placement in the middle PVT (but not the posterior PVT; Clavier and Gerfen, 1982). More recently, optogenetic self-stimulation was found to be supported when viral injections were made into the mouse PVT and fibers were placed in the nucleus accumbens (Lafferty et al., 2020), suggesting that stimulation of PVT projections to the accumbens can be rewarding. Support for a specific role of the anterior PVT in reward comes from studies in rats, which suggest that in fact inhibition of the anterior PVT promotes reward. Intracranial self-stimulation threshold, with the stimulating electrode targeted at the LH-medial forebrain bundle, is dose-dependently lowered by injection into the anterior PVT of the neuroinhibitory neuropeptide, cocaine- and amphetamine-regulated transcript peptide (CART), while injection of a CART antibody leads to the reverse effect (Choudhary et al., 2018). Similarly, time in the paired chamber in a real-time place preference paradigm is reduced by photoactivation of anterior PVT projections, both to the nucleus accumbens shell and the central nucleus of the amygdala (Do-Monte et al., 2017). It may be that animals experience reward from either inhibition of the anterior PVT (*via* its projections to the nucleus accumbens or amygdala) or stimulation of the middle PVT (also *via* projections to the nucleus accumbens). Conversely, in response to purportedly rewarding stimuli, including access to a female conspecific (for males) or thermoneutral zone, dopamine D2 receptor-expressing PVT neurons, which in the mouse are enriched in the posterior PVT, show a decrease in activity, as measured by calcium transients (Gao et al., 2020). Prior research in the mouse on single-unit excitation in the middle PVT has shown that about two-thirds of task-related neurons respond to outcomes that are both appetitive (water after water-restriction) and aversive (air puff or tail shock), while one third is specifically tuned to outcomes that are either appetitive or aversive and that these responses are proportionate with the intensity of the outcome (Zhu et al., 2018). The intermixing of these populations of cells may reflect the transition zone noted by other researchers between the anterior and posterior PVT (Dong et al., 2017; Gao et al., 2020). Altogether, these findings suggest that the PVT is both responsive to rewarding stimuli and itself can affect behavior reflecting reward and that while, to some extent, these responses may not be specific for reward or be clearly defined by PVT subregion, there may be populations of cells within the PVT that are more involved in this phenomenon.

### Depression—the PVT Participates in Depression-Like Behavior

Extremely limited evidence supports a role for the PVT in depression-like behavior. Following chronic forebrain expression of a mutation of a mitochondrial DNA polymerase, which has its highest accumulation in the PVT, female but not male mice show an increased number of depressive episodes, as measured by reduced wheel running, increased levels of corticosterone, increased sleep, and greater food intake (Kasahara et al., 2016). Further, genetic inhibition of PVT synaptic output by Cre-loxP-dependent expression of tetanus toxin similarly promotes these depression-like episodes (Kasahara et al., 2016). Interestingly, seemingly opposite effects have been reported more recently by this same group. Chronic presynaptic inhibition of PVT neurons by tetanus toxin in female mice was found to reduce immobility time in a forced swim test, while long-term chemogenetic activation of the PVT increased hypoactivity as measured by reduced wheel running (Kato et al., 2019). Short-term chemogenetic modulation did not affect immobility time in a forced swim test or tail suspension test (Kato et al., 2019). Thus, PVT activity appears to participate in depression-like behavior, but the direction of these effects and the subregions and pathways through which this occurs remain to be characterized.

### Anxiety—the Posterior PVT Plays a Major Role in the Regulation of Anxiety-Like Behavior

While there is discrepancy even within the same studies, the PVT also appears to participate in anxiety-like behavior, although findings on the direction of these effects are not always in agreement. In examining the PVT overall, optogenetic stimulation of PVT projections to the central amygdala in mice is found to reduce or leave unaffected time spent in the open arms of an elevated plus-maze (Chen and Bi, 2019; Pliota et al., 2020) while inhibition of this pathway following stress somewhat increases it (Pliota et al., 2020), indicating that the PVT-to-amygdala pathway functions to promote anxiety-like behavior. Stimulation of the posterior half of the PVT generally recapitulates these findings. Microinjection into the rat posterior PVT of the neurostimulatory neuropeptide, orexin, reduces time and number of entries into the open arms of an elevated plus-maze (Li et al., 2010b; Heydendael et al., 2011) and the number of visits to the center of an open field (Li et al., 2010a); conversely, an orexin receptor antagonist reduces the latency to enter the social interaction zone in a social interaction test (Dong et al., 2015) and, following foot shock, increases the time and number of entries into the open arms of an elevated plus-maze (Li et al., 2010b). On the other hand, inhibition of rat posterior PVT neuronal activity *via* microinjection of the GABA agonists, baclofen and muscimol, similarly reduces time and entries into the open arms of an elevated plus-maze (Barson and Leibowitz, 2015). Thus, the direction of the effects of posterior PVT activity on anxiety-like behavior may depend on the specific population of cells that is affected. While support for a role of the anterior PVT in anxiety-like behavior is less robust, some studies have nevertheless demonstrated this connection. Neither photostimulation of the rat anterior PVT on its own nor its projections to the nucleus accumbens shell affects time spent in the center of an open field, but this behavior is reduced by photostimulation of the projections to the central amygdala (Do-Monte et al., 2017). Similarly, neither GABAergic inhibition of the rat anterior PVT nor photostimulation of the mouse anterior PVT-to-accumbens pathway affects time in the open arms of elevated plus maze (Barson and Leibowitz, 2015; Cheng et al., 2018). On the other hand, photostimulation of this pathway does increase time spent feeding in a novelty-suppressed feeding test and tends to increase time spent in the light chamber of a light-dark box (Cheng et al., 2018). These results suggest that, under limited circumstances, the anterior PVT may also participate in anxiety, with its projections to the amygdala promoting, and to the accumbens inhibiting, anxiety-like behavior. Overall then, the posterior PVT demonstrates a robust role in anxiety-like behavior, generally promoting but also suppressing this behavior, while the anterior PVT makes a more limited contribution, similarly promoting and suppressing this behavior *via* separate neural pathways.

### Stress—the Posterior PVT Plays a Greater Role Than the Anterior PVT in the Response to Stress

A large body of evidence has connected the PVT with stress, with studies nearly uniformly demonstrating that, while the PVT responds across its antero-posterior axis to a range of purported stressors, the posterior PVT is more responsive than the anterior PVT to these stimuli. Levels of c-Fos in the whole PVT of both the rat and mouse are increased following withdrawal from alcohol (Knapp et al., 1998; Smith et al., 2019), although there is clear fluctuation in these levels throughout the withdrawal period (Smith et al., 2019). Similarly, levels of c-Fos in the mouse PVT are increased following exposure to an elevated plus-maze or foot shock (Pliota et al., 2020), and levels in the rat middle PVT are increased following a forced swim test (Zhu et al., 2011). While population calcium signaling in the rat is increased in both the anterior and posterior PVT following foot shock (Choi et al., 2019), levels of c-Fos are increased to a greater extent in the rat posterior compared to anterior PVT following noxious mechanical stimulation (Bullitt, 1990) and, in the obese Zucker rat, they can be identified at an earlier time-point following a period of food deprivation (Timofeeva and Richard, 2001). Similarly, levels of c-Fos are increased in the posterior but not anterior or middle PVT of the rat by acute restraint stress following chronic intermittent cold stress (Bhatnagar and Dallman, 1998). Moreover, calcium transients in dopamine D2 receptor-expressing PVT neurons, which are enriched in the mouse posterior PVT, are increased by aversive stimuli, including foot shock and tail suspension (Beas et al., 2018; Gao et al., 2020), and calcium events in a subset of cells in the mouse posterior PVT occur as a phasic response to footshock (Pliota et al., 2020). One exception to the greater response of the posterior PVT comes from a study that found that the anterior but not middle or posterior PVT showed elevated levels of c-Fos in mice after a novelty-suppressed feeding test, compared to mice exposed to a novel object or left naïve (Cheng et al., 2018). In light of the role of the anterior PVT in arousal, however (see “Arousal”), it may be that this test reflects differences in arousal more than it reflects stress. Overall then, the evidence as a whole supports a greater role for the posterior PVT in the response to stress.

The functional role of the posterior PVT appears to be a suppression of the stress response following chronic but not acute stressors, as demonstrated in a series of studies by Bhatnagar and colleagues. Lesioning of the rat posterior PVT blocks adaptation of the hypothalamic-pituitary-adrenal (HPA) axis (adrenocorticotropic hormone (ACTH) and corticosterone) to restraint stress, following repeated exposures to this stressor (Bhatnagar et al., 2002). Similarly, this same treatment blocks the reduction in amplitude in core body temperature rhythms after novel restraint stress in chronically cold-stressed rats but not in rats with no history of chronic stress (Bhatnagar and Dallman, 1999), and it increases the duration and height of burying an aversive stimulus in a conditioned defensive burying paradigm in chronically restraint-stressed rats but not in stress-naïve rats (Bhatnagar et al., 2003). This ability of the posterior PVT to inhibit the facilitation of the HPA axis to a novel stressor in chronically stressed rats appears to be due in part to orexin afferents from the LH. The facilitation of the HPA response to acute restraint stress is blocked by injection into the posterior PVT of an orexin receptor antagonist before chronic swim stress but not before acute restraint stress (Heydendael et al., 2011). Thus, while studies with orexin microinjections suggest that the function of the posterior PVT is to promote anxiety (see “Anxiety”), these studies suggest that it also promotes adaptation of the HPA axis to chronic stress and dampens HPA and anxiety responses to chronic stress.

## Conditioned Affective Behavior

In tests of conditioned affective behavior, the PVT has been shown to involved in both appetitive and aversive behavior, with its neuronal activity changing in response to and influencing reward-seeking and fear retrieval (see below for details). While the anterior and posterior halves of the PVT both appear to be involved in these behaviors, their relative contributions to each behavior again demonstrate significant variation.

### Reward and Motivation—the Anterior and Posterior PVT Differentially Regulate Reward/Motivation

Research on conditioned reward and motivation suggests that while the entire PVT is affected by and can affect the behavioral response to reward-predictive cues, activation of the anterior PVT generally inhibits motivated or seeking behavior while activation of the posterior PVT may instead promote it (but see Otis et al., 2017, 2019). Population calcium signaling in both the mouse and rat PVT is increased by stimuli that, in a Pavlovian conditioning paradigm, indicate the delivery of outcomes that are both appetitive (water after water-restriction or sucrose) and aversive (air puff, tail shock, or foot shock; Zhu et al., 2018; Choi et al., 2019). Notably, however, while a conditioned stimulus signaling the delivery of a sucrose reward stimulates activity in both the anterior and posterior PVT, activity in the anterior PVT is a weak predictor of magazine approach behavior (Choi et al., 2019). This may be because individual neurons in the anterior PVT are not uniformly activated, as it has been shown with unit-recording electrodes that firing rate can be either increased or decreased in the anterior PVT to a cue predicting sucrose availability under an operant conditioning paradigm (Do-Monte et al., 2017). In contrast, population calcium signaling in the posterior PVT serves as a strong predictor of magazine approach behavior, being increased both to a stimulus signaling the delivery of sucrose and to the consumption of that sucrose (Choi et al., 2019). Similarly, while gene expression of c-*fos* is increased in the anterior but not posterior PVT to a food-predictive cue in rats that have attributed incentive salience to the cue (Flagel et al., 2011), protein levels of c-Fos under similar conditions are elevated in posterior PVT afferents to the nucleus accumbens (Haight et al., 2017). Thus, neuronal activity in response to a reward-predictive cue can be detected throughout the PVT, but endogenous activity in the posterior PVT appears to be a more robust predictor of approach behavior.

In contrast to research on endogenous neuronal activity, research using experimenter-induced changes in neuronal activity suggests that approach behavior is affected by the anterior rather than the posterior PVT. Cue-induced sucrose magazine entries are not affected by chemogenetic inhibition of both anterior and posterior rat PVT (Choi et al., 2019) and cue-induced lever-pressing for a sucrose reward is unaffected by pharmacological inhibition of the rat posterior PVT, using microinjection of the GABA agonist, muscimol (Do-Monte et al., 2017). On the other hand, cue-induced lever-pressing for sucrose during a session where this reward is omitted (which can be conceptualized as a first extinction session) is increased by pharmacological inhibition of the rat anterior PVT and suppressed by photoactivation at cue onset (Do-Monte et al., 2017). This effect occurs *via* projections to the nucleus accumbens shell, as the inhibition of lever-pressing from PVT photoactivation is recapitulated by photoactivation of the anterior PVT-to-nucleus accumbens shell pathway and reversed by its photoinhibition (Do-Monte et al., 2017). In contrast, photoinhibition of the anterior PVT-to-central amygdala pathway, like photoactivation of the anterior PVT-to-nucleus accumbens pathway, reduces cue-induced lever-pressing for sucrose during a reward omission session (Do-Monte et al., 2017). Thus, approach or seeking behavior is affected by both the anterior and posterior PVT, but the direction of the effects of their activation may depend on the specific cell types and pathways involved.

### Fear—the Posterior PVT Plays a Critical Role in Fear Retrieval

The PVT also shows a clear role in conditioned fear, particularly fear retrieval, with significant research on this topic focused on the posterior PVT. Neurons in the PVT show increased activity to a conditioned fear tone, but only starting more than 6 h following tone-shock pairings, and lasting at least 7 days. For example, in the rat PVT, more neurons show electrophysiological responses to a conditioned fear tone tested 24 h but not 2 h after conditioning, and levels of c-Fos are elevated in a fear retrieval test conducted 7 days but not 6 h following conditioning (Do-Monte et al., 2015). This same elevation in c-Fos is found when the mouse posterior PVT, rather than the whole PVT, is examined in a fear retrieval test conducted 24 h following fear conditioning (Penzo et al., 2015). It is also found 7 days following fear conditioning in rat prelimbic neurons that project to the middle-to-posterior PVT and in middle-to-posterior PVT neurons that project to the central nucleus of the amygdala (Do-Monte et al., 2015). Thus, neurons in the posterior PVT become activated during conditioned fear retrieval and this occurs *via* a prelimbic-to-PVT-to central amygdala pathway. In turn, activation of the PVT and this pathway appears to facilitate the expression of conditioned fear following this same delayed timeline. Freezing to a conditioned tone during a fear retrieval test conducted 24 h but not earlier after conditioning is diminished by pharmacological inhibition, using microinjection of muscimol into the rat dorsomedial nucleus which spreads into the PVT (Padilla-Coreano et al., 2012). Similarly, fear retrieval 1 week after conditioning is suppressed by bilateral lesions made after fear conditioning of the rat posterior PVT (Li Y. et al., 2014), and it is reduced by chemogenetic inhibition of the rat middle-to-posterior PVT when tested with an operant food reward available (Choi and McNally, 2017). It should be noted, however, that studies have not uniformly found manipulation of the PVT to affect fear recall, as freezing to a conditioned fear tone is unaffected by chemogenetic inhibition of the rat anterior plus posterior PVT (Choi et al., 2019) and by microinjection of an orexin receptor antagonist into the rat posterior PVT (Dong et al., 2015). Despite this, there is solid evidence that activity in the prelimbic-to-PVT-to central amygdala pathway can promote the expression of conditioned fear. Fear retrieval is reduced by optogenetic silencing of prelimbic projections to the rat middle-to-posterior PVT (Do-Monte et al., 2015) and mouse PVT afferents to the central amygdala (Chen and Bi, 2019). Similarly, it is reduced by chemogenetic inhibition of mouse central amygdala-projecting PVT neurons (Penzo et al., 2015). In contrast, expression of conditioned fear is facilitated by photostimulation of mouse PVT afferents to the central amygdala (Chen and Bi, 2019). Collectively, the literature suggests that the PVT is recruited during consolidation of conditioned fear memory and, acting in response to prelimbic afferents and itself acting *via* the central amygdala, serves to promote the expression of conditioned fear.

## Conclusions and Future Directions

While much progress has been made in delineating the functional contribution of the PVT to emotional processing, some of the data as a whole remain equivocal, and, as such, many outstanding questions remain. The contribution of the specific PVT subregions does vary, based on the particular behavioral test employed and even within the same assay, suggesting that the exact role of the PVT may depend less on subregion and more on cell type. Very recent research has demonstrated the existence of antero-posterior gradients of two well-defined neuronal subtypes (Gao et al., 2020) and it is very likely that a similar pattern of distribution also exists for other neuronal subtypes within the PVT. Future studies are needed to develop efficient strategies to gain genetic access to these neuronal subtypes. The functional role of distinct PVT afferents and efferents in the regulation of specific behavioral tests of unconditioned and conditioned affective behavior are needed, and the application of genetic techniques for cell-type-specific monitoring and manipulation in the PVT will allow for rigorous testing of these questions. Of note, the discovery that many PVT projections are highly collateralized (Dong et al., 2017) has major implications for the design and interpretation of these tests. Future studies should involve well-controlled experiments for projection-specific manipulations, involving optogenetic and chemogenetic approaches, since the majority of PVT neurons project to the nucleus accumbens shell and provide collaterals to other regions, such as the BNST and central nucleus of the amygdala (Dong et al., 2017). Given the heterogeneity of PVT responses and effects on emotionally salient stimuli, future experiments should be designed to test the role of specific PVT cell types and connections in the processing of salient stimuli and subsequent behavioral output. It is especially crucial to understand how these different components of the PVT circuit (neuronal subtypes and afferent and efferent connections) act to encode distinct features of an explored environment to generate emotional fight-or-flight responses.

On the whole, however, evidence supports a strong role for the PVT in multiple aspects of emotional processing, demonstrating that it is both responsive to and itself affects both arousal and valence (Figure 1). The PVT, particularly the anterior PVT, can through its neuronal activity promote arousal, both as part of the sleep-wake cycle and in response to novel stimuli. The PVT is also involved in reward, being both responsive to rewarding stimuli and itself affecting behavior reflecting reward, likely *via* specific populations of cells distributed throughout its subregions. Similarly, PVT neuronal activity appears to affect depression-like behavior, through yet undefined subregions and cell populations. The posterior PVT in particular also demonstrates a role in anxiety-like behavior, generally promoting but also inhibiting this behavior. While this subregion is also especially responsive to stressors, it appears to function to suppress the stress response following chronic stress exposure. Following conditioning, the posterior PVT again plays a major role in emotional behavior. Neuronal activity in response to a reward-predictive cue can be detected throughout the PVT, but endogenous activity in the posterior PVT is a robust predictor of approach or seeking behavior. Similarly, neuronal activity during conditioned fear retrieval is detected in the posterior PVT and its activation appears to facilitate the expression of conditioned fear. Much of this involvement of the PVT in arousal and valence has been shown to occur through the same general afferents and efferents; however, a detailed functional map of the PVT circuits that control emotional responses, particularly those involving the posterior PVT, remains elusive. Afferents from the hypothalamus affect the involvement of the PVT in arousal, anxiety-like behavior, and the response to stress, and afferents from the prelimbic cortex affect its involvement in the expression of conditioned fear. In turn, through efferents to the nucleus accumbens, the PVT affects arousal, reward-related behavior, anxiety-like behavior, and motivation, and through efferents to the amygdala, it affects these same behaviors as well as the expression of conditioned fear. Finally, the PVT also affects arousal through efferents to the infralimbic cortex. It may be that some of these projections originate from the same PVT cells, but that remains to be determined. Thus, while caveats exist and more work is required to define its exact role, the PVT, through its extensive connections with other prominent nuclei in the limbic system, appears to be an integral part of the emotional processing network.

## Author Contributions

JB, NM, and W-JG wrote and edited the manuscript. All authors contributed to the article and approved the submitted version.

## Conflict of Interest

The authors declare that the research was conducted in the absence of any commercial or financial relationships that could be construed as a potential conflict of interest.
